# Application Value of a 6-Type Classification System for Common Hepatic Artery Absence During Laparoscopic Radical Resections for Gastric Cancer: A Large-Scale Single-Center Study

**DOI:** 10.1097/MD.0000000000001280

**Published:** 2015-08-14

**Authors:** Chang-Ming Huang, Rui-Fu Chen, Qi-Yue Chen, Jin Wei, Chao-Hui Zheng, Ping Li, Jian-Wei Xie, Jia-Bin Wang, Jian-Xian Lin, Jun Lu, Long-Long Cao, Mi Lin

**Affiliations:** From the Department of Gastric Surgery (C-MH, R-FC, Q-YC, C-HZ, PL, J-WX, J-BW, J-XL, JL, L-LC, ML); and Department of CT/MR, Fujian Medical University Union Hospital, Fuzhou, Fujian Province, China (JW).

## Abstract

The common hepatic artery (CHA) is an important blood vessel that must be vascularized during D2 lymphadenectomies for gastric cancer. When the CHA is absent, the risk of vascular injury increases.

To explore the anatomic classification of CHA absence and its application value in laparoscopic radical resections for gastric cancer.

Clinical data were collected prospectively from 2170 gastric cancer patients from June 2007 to December 2013, and the data were analyzed retrospectively. The anatomy of CHA absence was assessed synthetically by combining preoperative CT scans and intraoperative images, which were classified according to the anatomy of replaced hepatic arteries (RHAs) and were grouped into the early-year group (2007–2011) and the later-year group (2012–2013) based on the year in which the operation was performed.

CHA absence was noted in 38 cases (1.8%) and was classified into 6 types: type I (replaced CHA [RCHA] from the superior mesenteric artery [SMA] with retropancreatic course, 28), type II (RCHA from the SMA with circumambulated course, 1), type III (RCHA from the aortic artery, 1), type IV (replaced left hepatic artery [RLHA] from the left gastric artery [LGA] and replaced right hepatic artery [RRHA] from the SMA, 5), type V (RLHA from the LGA and RRHA from the celiac artery, 2), and type VI (RLHA from the aberrant gastroduodenal artery and RRHA from the SMA, 1). Of the 38 cases, 17 cases (44.7%) belong to the early-year group, and 21 cases (55.3%) belong to the later-year group. The vascular injury rate was significantly lower in the later-year group than in the early-year group (4.8% [1/21] vs 41.2% [7/17], *P* = 0.005]. Additionally, the alanine aminotransferase (ALT), aspartate aminotransferase (AST), and total bilirubin (TBIL) values were significantly lower in the later-year group than in the early-year group on postoperative day 3 (all *P* < 0.05).

A 6-type anatomic classification system can be used to demonstrate variations in features resulting from CHA absence in detail. Knowledge regarding a patient's classification is helpful for surgeons, and vascular injury and liver function damage may be reduced in patients who are properly classified prior to surgery.

## INTRODUCTION

The normal common hepatic artery (CHA) is derived from the celiac axis (CA), runs superior to the pancreas and anterior to the portal vein (PV), and is called the proper hepatic artery (PHA) after giving rise to the gastroduodenal artery (GDA). At the porta hepatis, the PHA separates into the right and left hepatic artery.^1^ The CHA is a pivotal vessel that must be completely dissected and skeletonized during suprapancreatic lymph node (LN) dissections for laparoscopic gastrectomies. CHA absence is noted occasionally during surgery. If this anatomic variation is not fully recognized by surgeons, vascular injury always occurs. According to Gray's anatomy, 40th ed,^1^ a CHA absence was defined as a CHA absence that directly arises from the CA, and a replaced hepatic artery (RHA) is a vessel that does not originate from an orthodox position and provides the sole supply to that lobe. The incidence of CHA absence is approximately 1.4% to 6.5%, as reported in the literature.^2–6^ However, the anatomical consequences of CHA absence are not yet completely understood. Although it was reported that the assessment of anatomic variations in perigastric vessels, such as the CHA, by preoperative computed tomography (CT) could reduce intraoperative bleeding;^7,8^ CHA absence has not yet been anatomically classified in such a way that assessment through preoperative CT could be conveniently utilized to reduce vascular injury risk during laparoscopic radical resections for gastric cancer. Herein, we report the first anatomical description of CHA absence during laparoscopic radical resections for gastric cancer through a large cohort study, and we suggest the use of a 6-type anatomic classification system for the determination of CHA absence. The use of this system will help increase our knowledge and ability to identify anatomic variations so that surgeons can reduce vascular injury.

## METHODS

### General Information

Beginning in June 2007, the Department of Gastric Surgery at Fujian Medical University Affiliated Union Hospital established a video database of laparoscopic surgeries for gastric cancer. Intraoperative data regarding anatomical variations in perigastric vessels were prospectively collected and retrospectively analyzed. Until December 2013, the video database included data on 2170 patients from Fujian Province, China, who underwent laparoscopic radical resections for gastric cancer. Preoperative 64-slice spiral CT examinations (Discovery CT750HD) were performed for all of the patients. The scans ranged from the diaphragmatic dome to the lower margin of the pubic bone. All of the patients underwent D2 lymphadenectomies during their gastrectomies, and the arteries above the superior border of the pancreas, such as the PV, RHA, splenic artery (SA), and left gastric artery (LGA), were revealed during each suprapancreatic LN dissection.

Anatomic variations in anatomic perigastric vessels were evaluated by 2 radiologists using preoperative CT images and by 2 surgeons using operative videos to identify patients with CHA absence. The original scans were 3-dimensionally reconstructed to confirm each assessment (however, this reconstruction was not conducted in 2 cases because the thin-slice scanning method was not available when CT scanning was first introduced in our hospital). The findings obtained from the 3-dimensional (3D) images were identical to the intraoperative findings. The anatomic variations in CHA absence were classified according to the RHA anatomy. Since 2012, on the basis of a solid understanding of the anatomic classification system, the anatomic variation “CHA absence” has been preoperatively evaluated by the surgeon via the 3D images to plan the operation such that vascular injury can be reduced. Therefore, according to the year in which the operation was performed, patients with CHA absence were grouped into the early-year group (2007–2011) and the later-year group (2012–2013). The intraoperative and postoperative data were retrospectively analyzed. LN classification was performed according to the Japanese classification of gastric carcinoma, 3rd English edition,^9^ and tumor staging was performed according to the 7th edition of the Union for International Cancer Control (UICC) tumor, node, metastasis staging system.^10^ Liver function changes were evaluated by examining alanine aminotransferase (ALT), aspartate aminotransferase (AST), and total bilirubin (TBIL) values. Written informed consent from all patients was obtained for the publication of this report and any accompanying images, and our institutional review board approved the study.

### Surgical Procedure

Suprapancreatic LN dissection was performed in all patients according to the Japanese gastric cancer treatment guidelines.^11^ The suprapancreatic LN dissection sequence was as follows: No. 11p → No. 7, 9 → No. 8a → No. 5 → No. 12a. Under normal conditions, dissection of the No. 8a LNs can be performed along the CA or GDA toward the CHA. When the CHA was absent, LNs anterior to the PV were regarded as No. 8a LNs at the moment after the dissection of the No. 11p LNs. Dissection of the No. 8a LN was performed anterior to PV, directly. First, the initial PV region was exposed with an ultrasonic scalpel in the retropancreatic space. Dissection was performed along the CA from the initial PV region. Subsequently, the left gastric vein (LGV) and LGA were vascularized and divided at their roots between the clips until the No. 7 and No. 9 LN dissections were accomplished. Second, the surgeon used an ultrasonic scalpel, with its nonfunctional surface oriented toward the PV, to carefully dissect along the anatomical space on the surface of the PV toward the direction of the duodenum until reaching the GDA or RHA origin; thus, the No. 8a LN dissections were complete.

### Statistical Analyses

All statistical analyses were performed using the Statistical Package for the Social Sciences 19.0. The measurement data are presented as the mean ± SD. Chi-square or Fisher exact tests were used to compare categorical variables, and the unpaired Student's *t*-test was used to compare continuous variables, as appropriate. The Kaplan–Meier method was used for survival analysis. *P* < 0.05 was considered to be statistically significant.

## RESULTS

### Anatomic Classification of CHA Absence

Among the 2170 gastric cancer patients, there were 38 cases of CHA absence; thus, the incidence rate was 1.8%. Because of the different RHA anatomies, the anatomy of CHA absence was classified into 6 types, as follows:

Type I: The replaced common hepatic artery (RCHA) arose from the superior mesenteric artery (SMA) and ran across the posterior side of the pancreas in 28 cases (1.29%, 28/2170).

**FIGURE 1:**
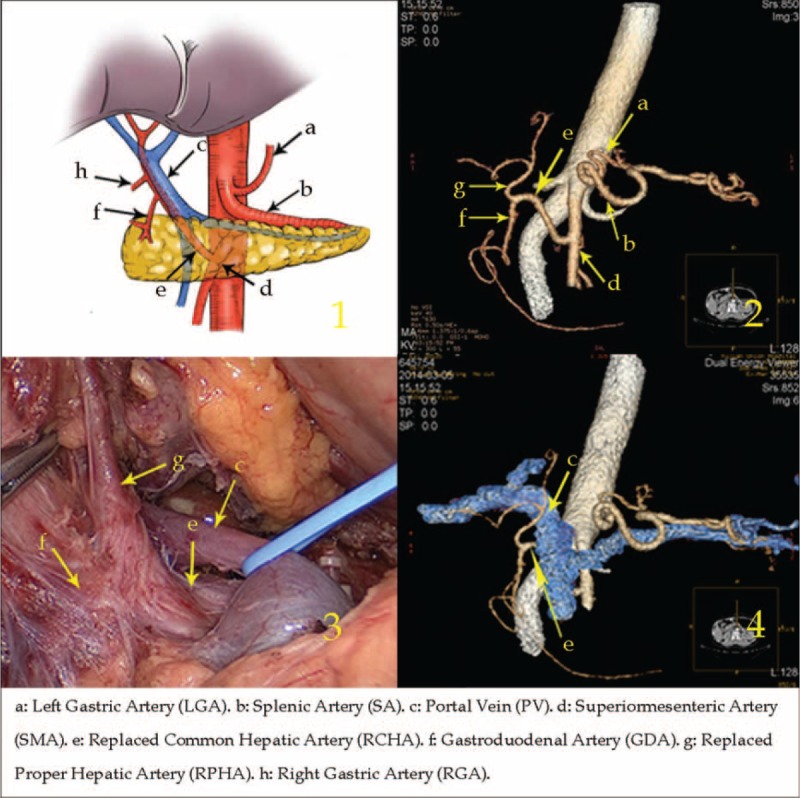
Type I: The replaced common hepatic artery arose from the superior mesenteric artery and ran across the posterior side of the pancreas.

In this type, after arising from the SMA, the RCHA ran to the upper right, behind the pancreas and PV. Then, the RCHA branched off the GDA and replaced proper hepatic artery (RPHA) when it laid adjacent to the superior region of the duodenum (Figure 1).

Type II: The RCHA arose from the SMA with a circumambulated approach anterior to the pancreatic head in 1 case (0.05%, 1/2170).

**FIGURE 2:**
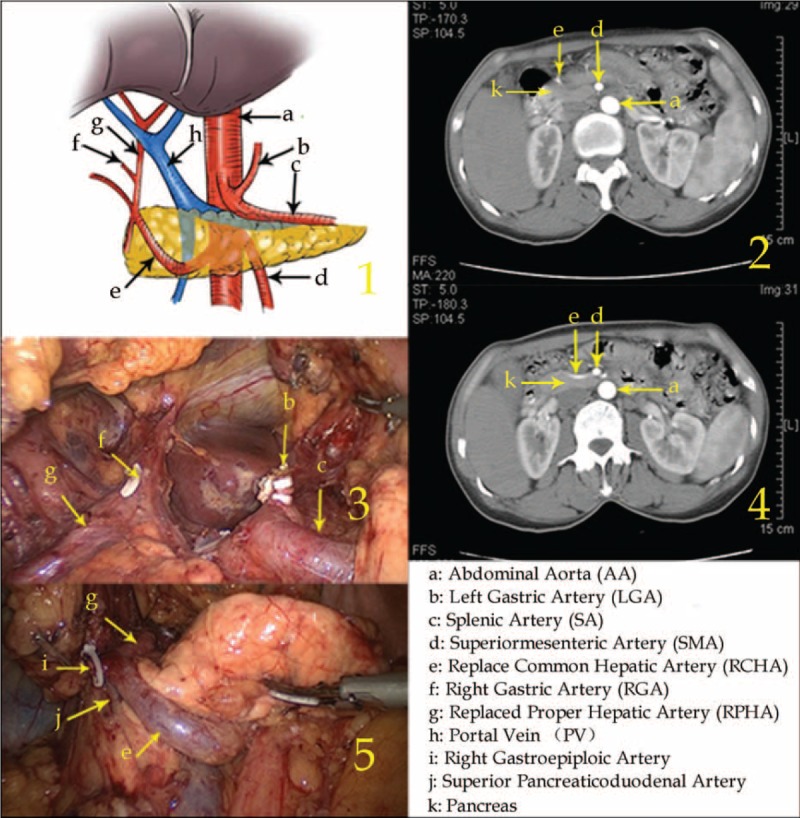
Type II: The replaced common hepatic artery arose from the superior mesenteric artery with a circumambulated approach anterior to the pancreatic head.

In this type, after arising from the SMA, the RCHA ran downward toward the inferior border of the pancreas, then surrounded the pancreatic head in a “U” shape and subsequently ran to the upper right. When it reached the superior border of the pancreas and the duodenal medial wall junction, the RCHA branched off the RPHA, which ran into the hepatoduodenal ligament located at the right side of the PV and further bifurcated into the replaced left hepatic artery (RLHA) and replaced right hepatic artery (RRHA) (Figure 2).

Type III: The RCHA arose from the aortic artery in 1 case (0.05%, 1/2170).

**FIGURE 3:**
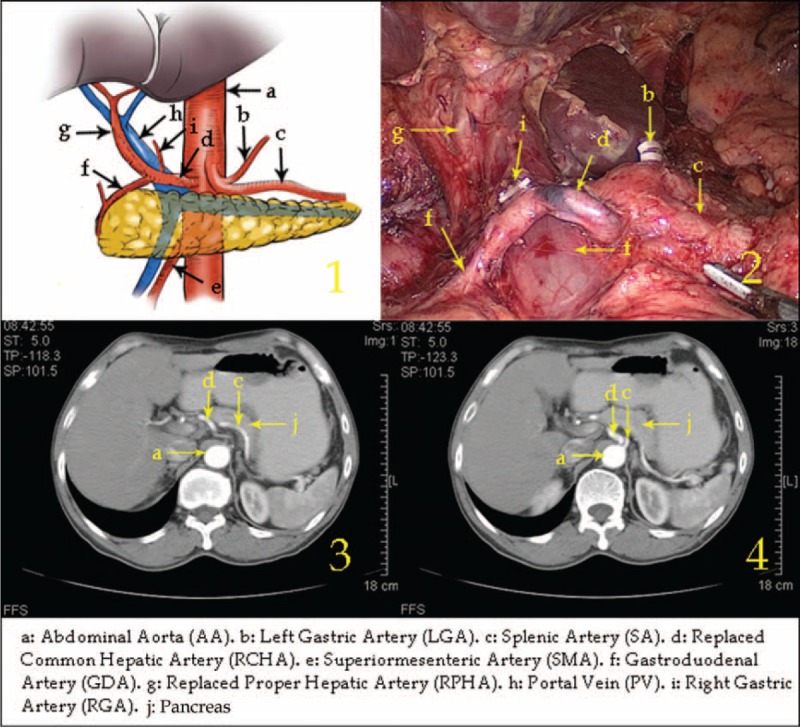
Type III: The replaced common hepatic artery arose from the aortic artery.

The RCHA arose from the aortic artery, passed in front of the PV, and bifurcated into the GDA and the RPHA on the right side of the PV, and the latter split into left and right branches. Additionally, the right gastric artery (RGA) originated from the RCHA in front of the PV (Figure 3).

Type IV: The RLHA arose from the LGA and the RRHA arose from the SMA in 5 cases (0.23%, 5/2170).

**FIGURE 4:**
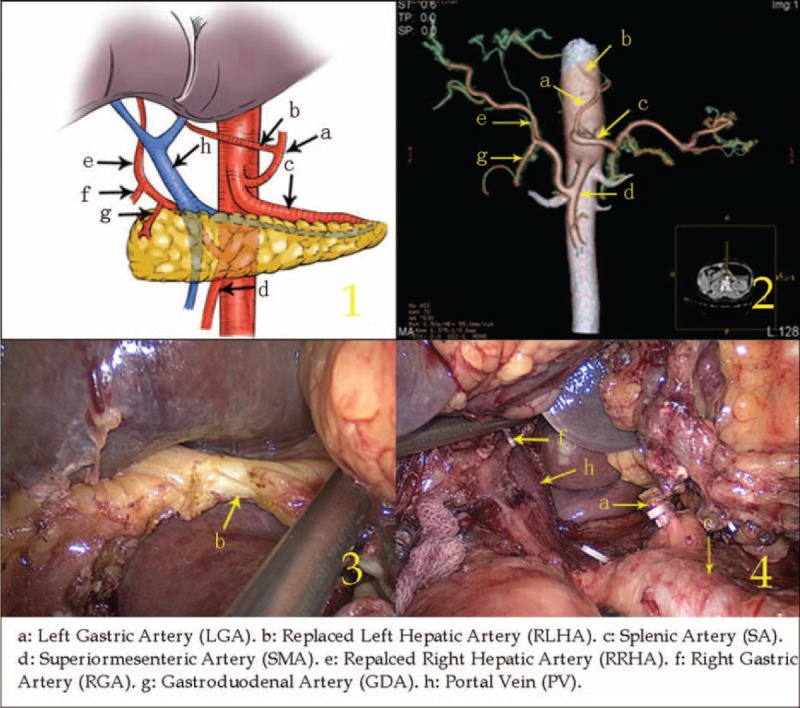
Type IV: The replaced left hepatic artery arose from the left gastric artery, and the replaced right hepatic artery arose from the superior mesenteric artery.

In this type, the RLHA was in the lesser omentum and supplied blood to the left liver lobe. The RRHA ran behind the PV after arising from the SMA and supplied blood to the right liver lobe, branching off to form the GDA in the suprapancreatic border horizon (Figure 4).

Type V: The RLHA arose from the LGA and the RRHA arose from the CA in 2 cases (0.09%, 2/2170).

**FIGURE 5:**
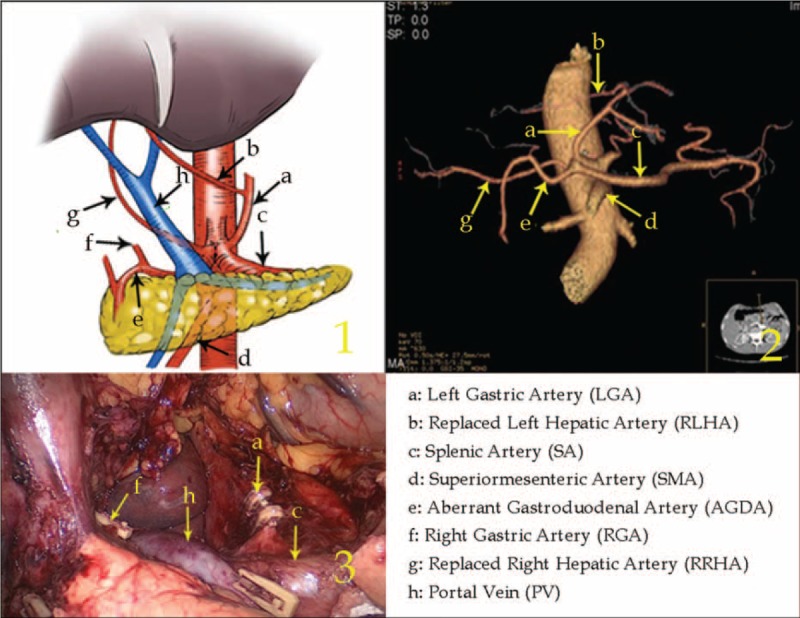
Type V: The replaced left hepatic artery arose from the left gastric artery, and the replaced right hepatic artery arose from the celiac artery.

The RLHA was in the lesser omentum supplying blood to left lateral liver lobe, and the RRHA passed behind the PV on the superior border of the pancreas, which supplied blood to the right liver lobe. The aberrant GDA arose from the CA and ran between the pancreatic head and duodenum. It then branched off to form a small artery, which supplied the left medial liver lobe (Figure 5).

Type VI: The RLHA arose from the aberrant GDA and the RRHA arose from the SMA in 1 case (0.05%, 1/2170).

**FIGURE 6:**
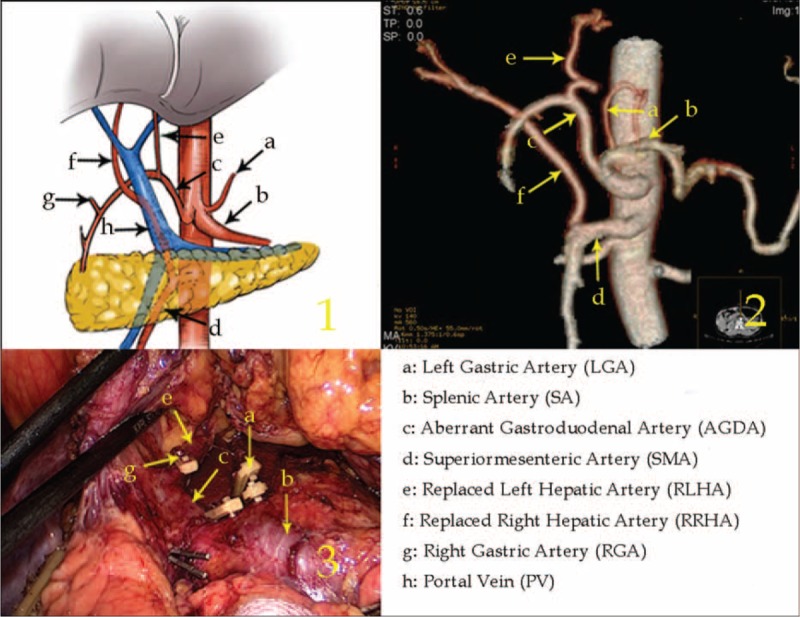
Type VI: The replaced left hepatic artery arose from the aberrant gastroduodenal artery, and the replaced right hepatic artery arose from the superior mesenteric artery.

The aberrant GDA arose from the CA and ran along the lesser curvature, branching off the RLHA when it was adjacent to the duodenal posterior wall. The RGA branched off from the RLHA. The RRHA arose from the SMA and passed behind the PV, supplying the right liver lobe. Meanwhile, a thin LGA branched from the CA on the left side of the aberrant GDA (Figure 6).

### Clinicopathologic Characteristics

The clinicopathologic characteristics of the patients with CHA absence are shown in Table 1. The 38 patients consisted of 27 men and 11 women. The mean age was 61.6 ± 12.4 years. Based on the operation year, 17 (44.7%) cases were classified into the early-year group (from 2007 to 2011), and 21 (55.3%) cases were classified into the later-year group (from 2012 to 2013). The incidence rate of CHA absence was similar between the 2 groups [1.5% (17/1094) vs 2.0% (21/1076), *P* = 0.480]. There were no statistically significant differences between the 2 groups with respect to clinicopathologic characteristics, including sex, age, body mass index, location of the tumor, tumor depth, N stage, tumor, node, metastasis stage, grade of differentiation, resection extent, and reconstruction type (all *P* > 0.05).

**TABLE 1:**
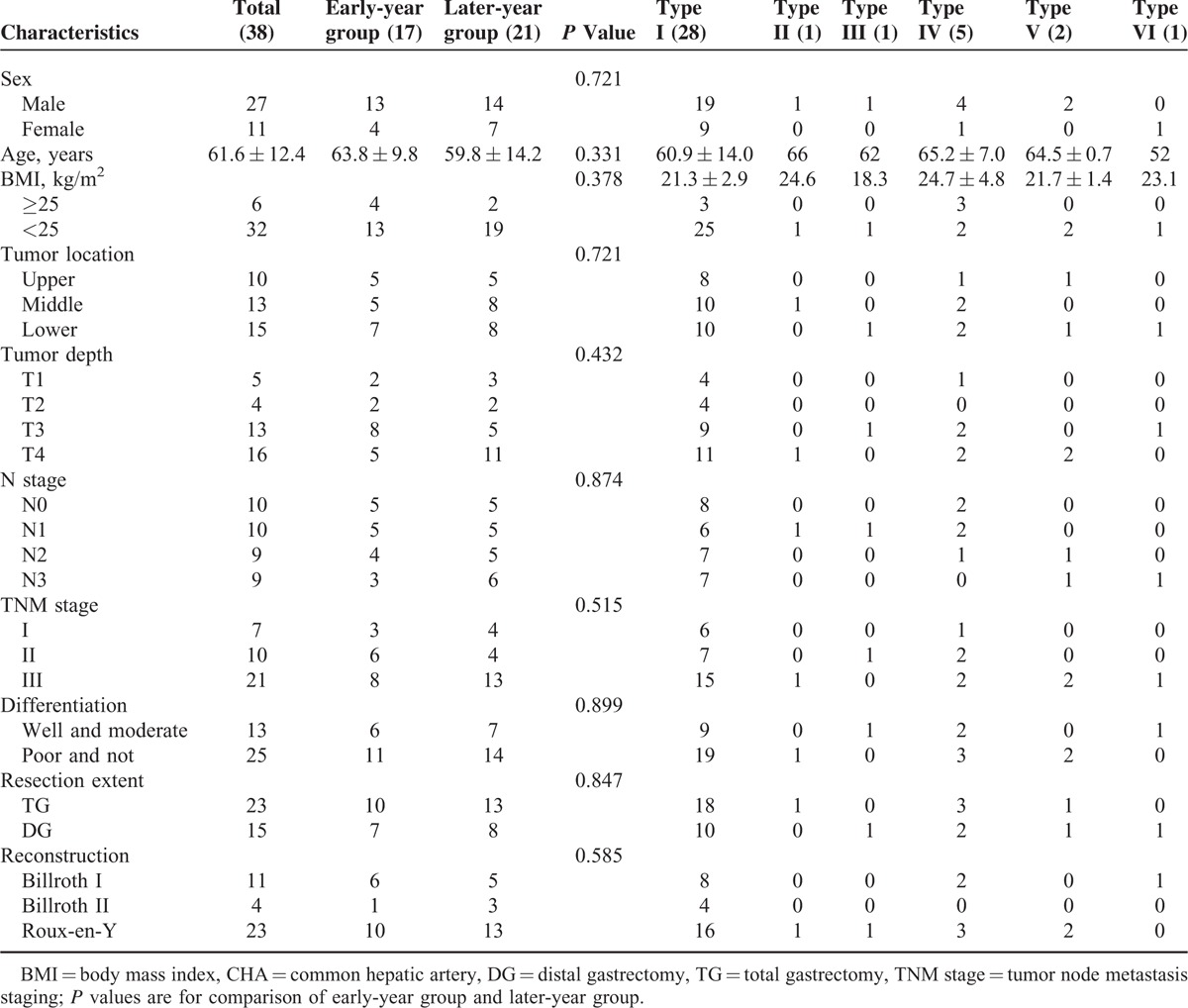
Clinicopathologic Characteristics About Patients With CHA Absence

### Intraoperative and Postoperative Characteristics

**FIGURE 7:**
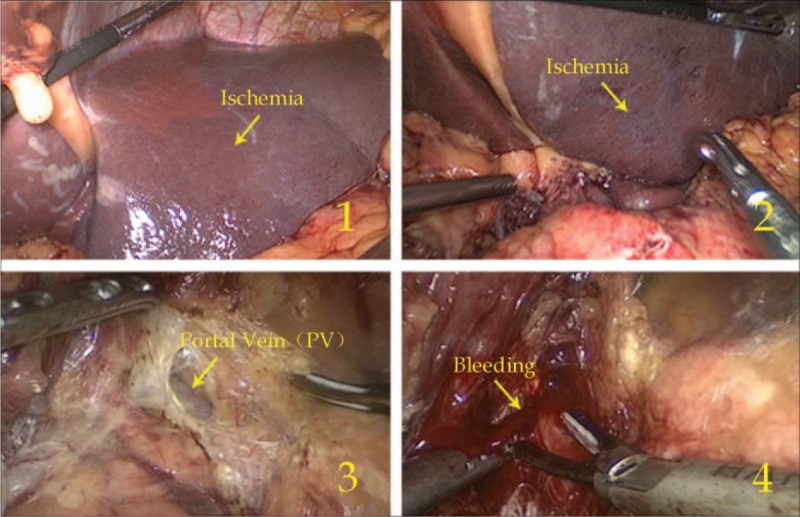
Ischemia of the left liver lobe (1, 2) and PV injury (3, 4).

Surgeries were performed successfully on all 38 patients. As shown in Table 2, the total operation time, total blood loss, blood loss during suprapancreatic lymphadenectomy, bowel function recovery time, and duration of hospital stay were similar between the 2 groups (all *P* > 0.05). However, time of suprapancreatic lymphadenectomy and time to first ambulation were shorter in the later-year group than in the early-year group (all *P* < 0.05). Vascular injury occurred in 8 patients during surgery, with 1 case of PV injury and 7 cases of RLHA injury. In the early-year group, 1 case of PV injury and 6 cases of RLHA injury were noted; PV injury resulted in a conversion to open surgery for PV repair because of the difficulty in achieving hemostasis during laparoscopy, and RLHA injury led to left liver ischemia in 4 cases with postoperative liver dysfunction (Figure 7). In the later-year group, however, only 1 case of RLHA injury was noted. The vascular injury rate was significantly lower in the later-year group than in the early-year group [4.8% (1/21) vs 41.2% (7/17), *P* = 0.005]. There were no postoperative deaths within 30 days. The incidence of postoperative complications was 13.2% (5/38), and there was no significant difference between the 2 groups [17.6% (3/17) vs 9.5% (2/21), *P* = 0.461]. Of the 5 cases with postoperative complications, 3 were in the early-year group. These complications included a duodenal stump fistula, a pancreatic fistula and pneumonia in 1 case and pneumonia alone in 2 cases. Two of the cases with postoperative complications were in the later-year group, including 1 case of abdominal infection and 1 case of abdominal infection plus pneumonia. According to the Clavien–Dindo grading system for complications,^12^ only duodenal stump fistula was classified as grade IIIa; the other complications were classified as grade II.

**TABLE 2:**
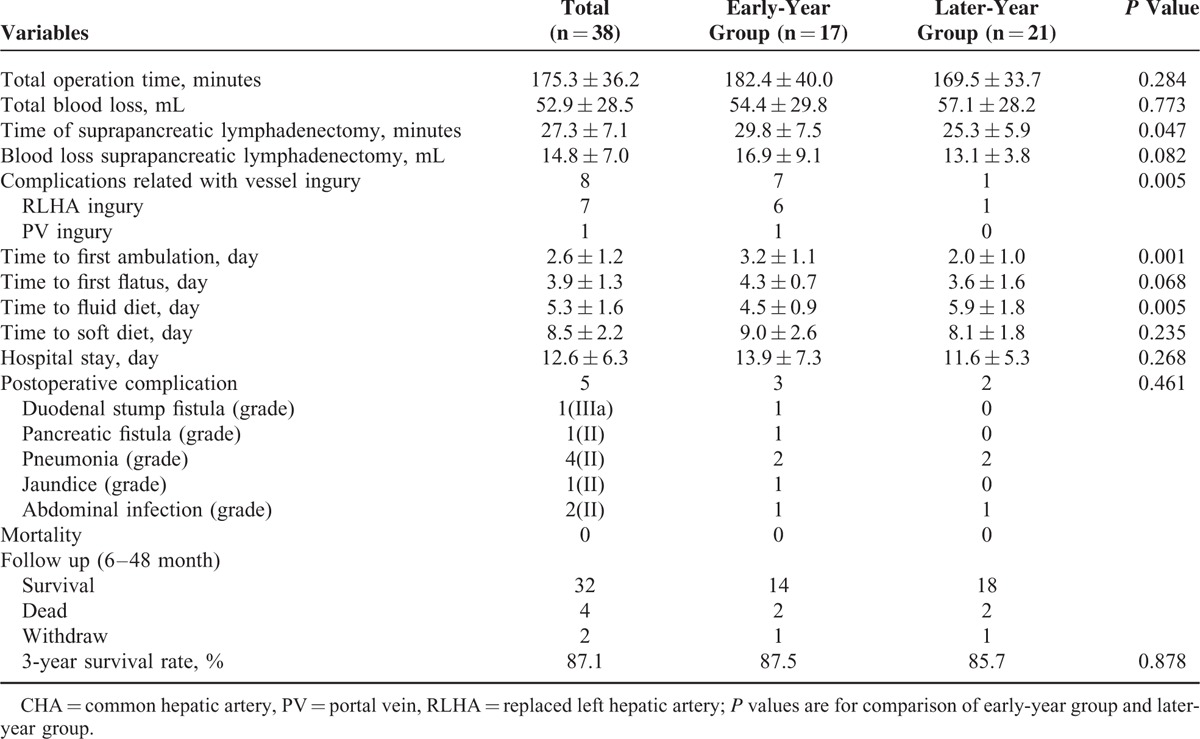
Intraoperative and Postoperative Information of Patients With CHA Absence

### LN Dissection

The LN dissection outcomes are shown in Table 3. The mean number of LNs retrieved from the 38 patients was 38.4 ± 14.8, with no significant difference between the 2 groups (36.7 ± 12.4 vs 40.3 ± 16.8, *P* = 0.462). The mean number of suprapancreatic LNs (Nos. 5, 7, 8a, 9, 11p, and 12a) retrieved from all patients was 13.2 ± 4.7, and this number was also similar between the 2 groups (*P* > 0.05).

**TABLE 3:**
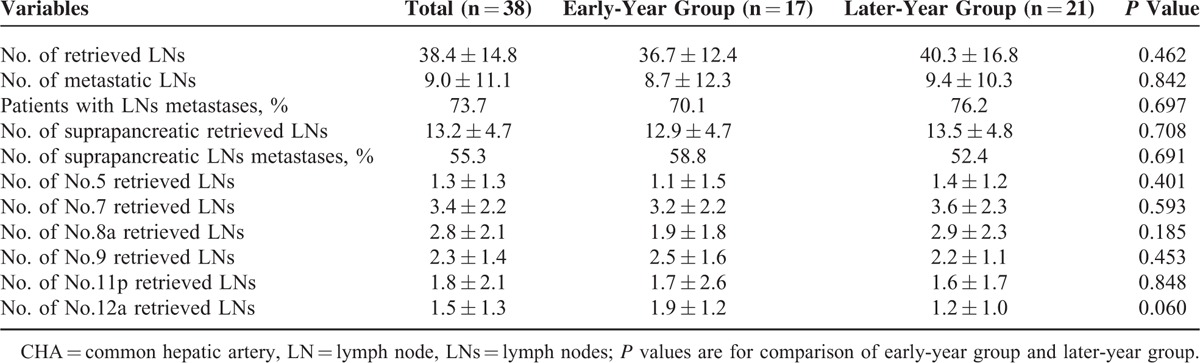
Outcome of LN Dissection About Patients With CHA Absence

### Postoperative Liver Function Changes

Table 4 shows the postoperative changes in ALT, AST, and TBIL values within 7 postoperative days (PODs). The preoperative ALT, AST, and TBIL values were similar between the 2 groups, as were the ALT, AST, and TBIL values on PODs 1, 5, and 7 (all *P* > 0.05). However, the ALT, AST, and TBIL values on POD 3 in the later-year group were significantly lower than those in the early-year group (all *P* < 0.05).

**TABLE 4:**
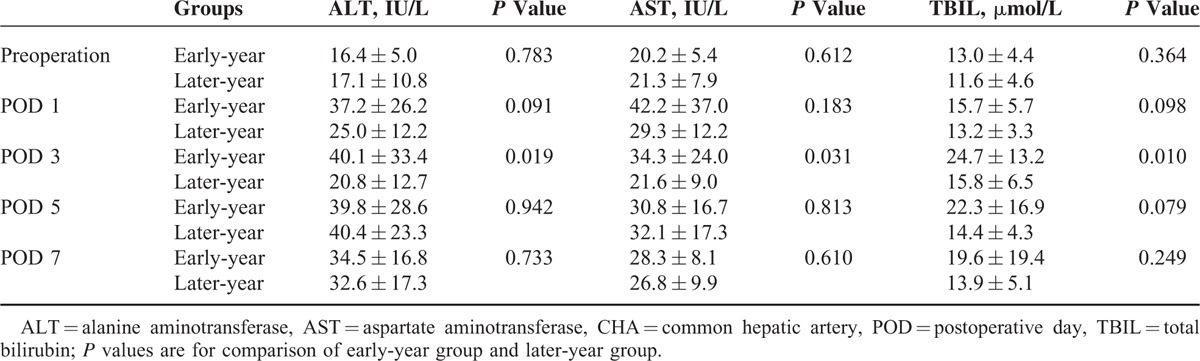
Mean ALT, AST, and TBIL Concentrations in Patients With CHA Absence

### Survival Analysis

Thirty six cases (94.7%) underwent postoperative follow-up. The follow-up duration ranged from 6 to 48 months (mean, 24 months), and the overall 3-year survival rate was 87.1%, with no significant difference between the early-year group and the later-year group (87.5% vs 85.7%, *P* > 0.05).

## DISCUSSION

Since laparoscopy-assisted distal gastrectomy was first reported by Kitano et al^13^ for the treatment of early gastric cancer in 1994, laparoscopic surgery has gradually become popular for radical gastrectomy for early gastric cancer cases. This approach has several advantages, such as minimal invasiveness, rapid gastrointestinal recovery, and short hospital stays.^14^ Its indication has been extended to advanced gastric cancer cases,^15^ and the extent of lymphadenectomy has developed from D1 to D2.^16^ However, LN dissection during laparoscopic radical resection for gastric cancer, especially suprapancreatic LN dissection, is technically difficult along the blood vessels because of the various anatomic variations in the perigastric vessels and the narrow visual field that is available with laparoscopy.^17^ Knowledge about the anatomic variations of the perigastric vessels, particularly the suprapancreatic vessels, is important so that vascular injury may be avoided during laparoscopic suprapancreatic LN dissections. To classify the variations in hepatic artery anatomy, Michels^3^ proposed a classic classification system in 1966, which served as a benchmark for all subsequent studies in this area. Many other classification systems have been derived from this classic system (Table 5).^4–6^ However, there are currently no classification systems that demonstrate the anatomic variations in CHA absence in detail. Further, the categories in the existing classification systems, which are based on autopsies, liver grafts or angiographies, cannot be accurately determined by preoperative CT scans and cannot be used to assess the potential vascular injury risk during laparoscopic radical resections for gastric cancer. The value of 3D-CT in the assessment of perigastric vessel variation has been confirmed;^18–20^ therefore, we propose a classification system for the anatomy of CHA absence based on preoperative CT scans and intraoperative images. The classification system includes 6 types, and 4 of these types are rare variants that were not reported in the Michels classification (Table 5). Knowledge regarding these anatomic classifications will allow surgeons to determine the type of CHA absence through preoperative 3-D CT images. If CHA absence is assessed and the type is defined preoperatively, the surgeon can evaluate the potential vascular injury risk during surgery and propose an optimal operation scheme as well as an alternative intraoperative plan to avoid vascular injury.

**TABLE 5:**
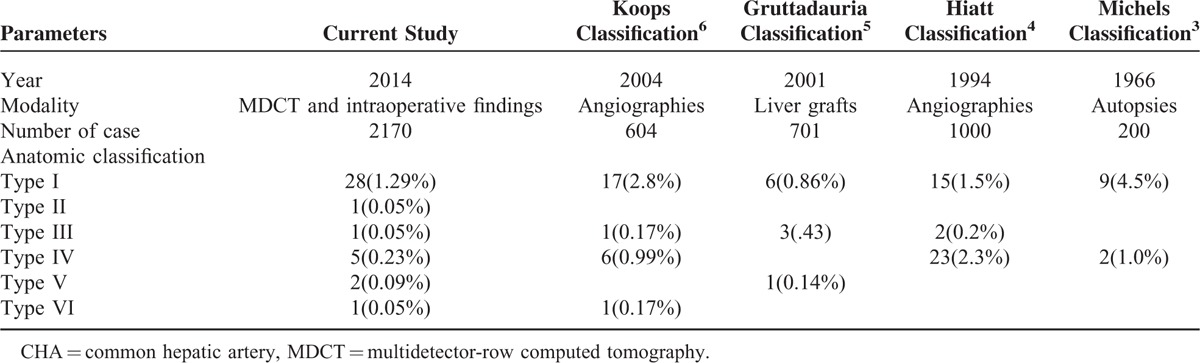
Classification and Previous Findings of Anatomy About CHA Absence

When operating on patients with CHA absence, the surgeon should avoid the PV and carefully explore other pancreatic areas to determine other arterial courses and branches, such as the LGA and GDA, with the aim of identifying and preserving the aberrant blood supply to the liver. For patients with different variations, however, the treatment method is somewhat different. For example, when a preoperative assessment indicates that the RCHA runs behind the PV (type I), the PV is directly exposed in the surgical field due to a loss of protection from the CHA. Junior surgeons may damage the PV and cause hemorrhage, which could even lead to a massive uncontrolled hemorrhage (Figure 7); therefore, when dissecting No. 8a LNs, the surgeon should shear gently with an ultrasonic scalpel and stretch the surrounding tissue appropriately to enter the correct space anterior to the PV, avoiding damage to the PV. If this damage occurs, it will be impossible for the surgeon to grasp the hemorrhagic area directly, which may enlarge the hole and cause a more severe hemorrhage.^21^ Minor bleeding can be handled through adequate gauze compression. If the bleeding is too serious to control via compression of the hemorrhagic area, the operation should be converted to open surgery for suture repair or PV reconstruction.^22^ If the RCHA arises from the SMA and a circumambulated approach anterior to the pancreatic head (type II) is preoperatively selected, the RCHA will be trifurcated into the right gastroepiploic artery, superior pancreaticoduodenal artery and RPHA while the GDA is absent; therefore, a lack of careful identification of these arteries during surgery will lead to incorrect resection. As previously reported, resection of the accessory LHA may cause left hepatic ischemia.^23^ Because the RLHA is the only artery supplying the left liver lobe, left hepatic ischemia can easily occur when the RLHA is resected, and severe complications, such as hepatic necrosis and hepatic failure, may occur postoperatively.^24–26^ Thus, the RLHA should be preserved as completely as possible during surgery. However, the injury rate of the RLHA reached 18.4% (7/38), and 85.7% (6/7) of these injuries occurred in the early-year group. This was because most of the RLHAs arose from the LGA and were in the lesser omentum (type IV and V), which was difficult to identify; if the anatomic variation was not recognized preoperatively, the surgeon often resected the LGA at its root, resulting in the accidental resection of the RLHA.^27^ Consequently, if this variation is determined preoperatively, the LGA should be dissected after it branches off the RLHA. In some cases, the individual RLHA arises from the aberrant GDA (type IV), which can easily be regarded as the RGA and accidentally resected; therefore, the surgeon should carefully separate and identify the vessels to preserve the RLHA. If the RLHA is severed, the liver function of the patient should be constantly monitored, and the patient should be administered liver protection therapy when necessary.^23^

In our study, the finding that the vascular injury rate was significantly lower in the later-year group compared with the early-year group is mainly due to the better understanding of the CHA absence anatomy; however, several confounding factors need to be considered, such as improvement in the proficiency of the surgeons and improvement in the facilities overall. Although 38 patients underwent multidetector-row CT prior to surgery, the proportion of patients with vascular injury was relatively high because the anatomic variations of CHA absence were overlooked when the practice of laparoscopic gastrectomy was adopted at our center. Because of the rare incidence of CHA absence, there were only 1 or 2 patients in 4 of the 6 categories. Additionally, the finding that there were no severe postoperative complications, such as hepatic failure or death, associated with RLHA excision may have been due to the small number of patients. However, we will continue to examine cases of CHA absence in the future.

In summary, a 6-type anatomic classification system can be used to classify the anatomic variation associated with CHA absence in detail. If the surgeon has an understanding of this system and ability to identify the anatomic variations, vascular injury and liver function damage can be reduced during laparoscopic radical resection for gastric cancer. This study was limited by the fact that it was a retrospective, nonrandomized clinical analysis, and a small number of patients with CHA absence were enrolled. Therefore, a prospective randomized study including more patients should be conducted to confirm our conclusions.
